# Modeling within and between Sub-Genomes Epistasis of Synthetic Hexaploid Wheat for Genome-Enabled Prediction of Diseases

**DOI:** 10.3390/genes15030262

**Published:** 2024-02-20

**Authors:** Jaime Cuevas, David González-Diéguez, Susanne Dreisigacker, Johannes W. R. Martini, Leo Crespo-Herrera, Nerida Lozano-Ramirez, Pawan K. Singh, Xinyao He, Julio Huerta, Jose Crossa

**Affiliations:** 1 Departamento de Energía, Universidad Autónoma del Estado de Quintana Roo, Chetumal 77019, Quintana Roo, Mexico; jaicueva@uqroo.edu.mx; 2 International Maize and Wheat Improvement Center (CIMMYT), Km. 45, Carretera México-Veracruz, Texcoco 56237, Edo. de México, Mexico; d.g.dieguez@cgiar.org (D.G.-D.); s.dreisigacker@cgiar.org (S.D.); johannes_martini@web.de (J.W.R.M.); l.crespo@cgiar.org (L.C.-H.); pk.singh@cgiar.org (P.K.S.); x.he@cgiar.org (X.H.); j.huerta@cgiar.org (J.H.); 3 Colegio de Postgraduados (COLPOS), Montecillos 56230, Edo. de México, Mexico

**Keywords:** synthetic hexaploid wheat (SHW), sub-genomes, epistasis, genomic prediction

## Abstract

Common wheat (*Triticum aestivum*) is a hexaploid crop comprising three diploid sub-genomes labeled A, B, and D. The objective of this study is to investigate whether there is a discernible influence pattern from the D sub-genome with epistasis in genomic models for wheat diseases. Four genomic statistical models were employed; two models considered the linear genomic relationship of the lines. The first model (G) utilized all molecular markers, while the second model (ABD) utilized three matrices representing the A, B, and D sub-genomes. The remaining two models incorporated epistasis, one (GI) using all markers and the other (ABDI) considering markers in sub-genomes A, B, and D, including inter- and intra-sub-genome interactions. The data utilized pertained to three diseases: tan spot (TS), septoria nodorum blotch (SNB), and spot blotch (SB), for synthetic hexaploid wheat (SHW) lines. The results (variance components) indicate that epistasis makes a substantial contribution to explaining genomic variation, accounting for approximately 50% in SNB and SB and only 29% for TS. In this contribution of epistasis, the influence of intra- and inter-sub-genome interactions of the D sub-genome is crucial, being close to 50% in TS and higher in SNB (60%) and SB (60%). This increase in explaining genomic variation is reflected in an enhancement of predictive ability from the G model (additive) to the ABDI model (additive and epistasis) by 9%, 5%, and 1% for SNB, SB, and TS, respectively. These results, in line with other studies, underscore the significance of the D sub-genome in disease traits and suggest a potential application to be explored in the future regarding the selection of parental crosses based on sub-genomes.

## 1. Introduction

Common wheat (*Triticum aestivum*) is an allohexaploid crop with three diploid sub-genomes, named A, B and D. The spontaneous hybridization of the A genome ancestor (*Triticum urartu* Tumanian ex Gandylian) and the B genome ancestor (*Aegilops speltoides* Tausch) formed the tetraploid species, *Triticum turgidum* L. (2n = 4x = 28, AABB). Another hybridization of *Triticum turgidum* L. with a single lineage of goat grass, *Aegilops tauschii* Coss, (2n = 2x = 14, DD) generated today’s hexaploid common (bread) wheat *T. aestivum* (2n = 6x = 42, AABBDD). The D genome shows less genetic variation than either the A or B genomes [1]. Common wheat was first domesticated in the Fertile Cresent, and spread from there to North Africa, Europe, and East Asia [2]. The International Maize and Wheat Improvement Center (CIMMYT) started to explore the potential of synthetic hexaploid wheat (SHW) in the 1980′s by crossing tetraploid durum wheat (DW, *Triticum turgidum* subsp. *durum* or *Triticum durum*) with *Ae*. *tauschii* accessions [3,4,5,6,7,8,9]. Many of these SHWs have shown resistance or tolerance to various biotic and abiotic stresses, indicating the potential of *Ae. tauschii* for breeding purposes [8,10].

Today, the use of molecular markers is common practice in plant breeding programs, such as in the context of marker-assisted selection, association studies [11,12,13], or genomic prediction [14,15,16]. With genomic selection illustrating its potential in animals, particularly in dairy cattle breeding, today’s most used additive genomic relationship matrix has originally been defined in the animal breeding literature by VanRaden [17]. For wheat, Bonnett [18] and Dreisigacker [19] presented empirical research on genomic-selection-enabled rapid cycling and reported increases in genetic gain over time compared to phenotypic selection.

For genomic prediction, additive and non-additive genetic effects can be estimated in statistical linear regression models based on (tens of) thousands of molecular markers using high-throughput genotyping platforms of target populations. For wheat, the inclusion of statistical non-additive effects in prediction models, either in the form of a general “non-additive relationship”, for instance a Gaussian kernel [20], or by modeling pairwise interaction effects between markers explicitly [21,22], has been shown to have the potential to increase predictive ability. A model with explicit pairwise interaction terms is equivalent to the use of Hadamard product of the additive relationship matrix, but correction terms may be required if one aims at a specific exact interaction effect model instead of an approximation [23,24].

When working with molecular markers in a context of quantitative genetics, for instance for genomic prediction, the hexaploid common wheat has usually been treated as diploid with 21 chromosomes, addressing the set of sub-genomes jointly without modeling a structural relation or separation of the three sub-genomes A, B, or D. However, the positions of molecular markers, and particularly their affiliation with the respective sub-genome, are usually known. This information would allow us to estimate additive and epistatic genetic covariances and effects at the level of sub-genomes. Santantonio [1] partitioned the additive and epistatic variances to the sub-genomes A, B, and D of wheat and predicted breeding values for each sub-genome. The authors determined the importance of inter-sub-genomic epistasis and concluded that estimating sub-genome breeding values will help breeders to better assess breeding goals by developing new strategies for the selection of additive effects and to exploit genomic epistasis.

In a recent study, Dreisigacker [19] applied hybrid prediction to the allopolyploidization event of SHW for wheat diseases, tan spot (TS), septoria nodorum blotch (SNB), and spot blotch (SB). The authors showed that prediction abilities were high when estimating the performance of untested SHWs, indicating that the method can guide the use of genetic resources available in gene banks.

In this study, we present results for various genome-based prediction models assessing the epistatic interaction within and between sub-genomes A, B, and D of SHW. We use the data of 443 synthetic SHWs that were genotyped and phenotyped for resistance to three different diseases in a previous study by Lozano-Ramirez [25,26]. In contrast to Dreisigacker [19], we do not use a hybrid prediction model including general and specific combining abilities of different parental groups—*durum* and *Ae. tuschii*— but we do use the genotypic data of SHW lines and split the effects into additive effects and additive interactions between the three sub-genomes.

## 2. Materials and Methods

A total of 443 SHW lines were generated by the CIMMYT Wheat Wide Crosses Program via the hybridization of 40 durum wheat (DW) parents, and 277 *Ae. tauschii* accessions were used in this study. The DW parents were involved in 1 to 54 crosses and the *Ae. tauschii* accessions were used in 1 to 7 crosses. The SHWs were selected from a larger collection of 1524 SHWs for their resistance to diseases such as Fusarium head blight, Septoria tritici blotch, rust, and acceptable phenology such as plant height and days to heading. Full details are given in Lozano-Ramirez [25,26].

### 2.1. Phenotypic Evaluations

The disease screening was carried out in a greenhouse at CIMMYT, El Batán, Mexico (19°31′ N, 98°50′ W, elevation 2249 m above sea level) in 2018–2019. All 443 SHW, along with the 40 DW parents, were evaluated for SB, TS, and SNB resistance at the seedling stage, while the *Ae. tauschii* accessions could not be screened due to their nature and growth as a wild species. The seed of SHW lines was vernalized to break down seed dormancy and to obtain an even germination. As described in Lozano-Ramirez [25,26] and Dreisigacker [19], the experiments were arranged in the greenhouse with 12 replicates for diseases TS and SNB, and 6 replicates for the disease SB, measured following the 1–5 ordinal lesion rating scale developed by Lamari and Bernier [27]. For each SHW entry, four plants were grown in plastic containers as experimental units to derive mean values used on the phenotypic model. The seedlings were grown under controlled conditions with an ambient temperature of 22–25/16–18 °C (day/night) and with a 16 h photoperiod [25,26]. Seedlings were inoculated at the two-leaf stage, when the second leaf was fully expanded, or two weeks after sowing.

### 2.2. Genotyping

Genomic DNA was extracted from the second leaf of 10-day-old seedlings of each line of the SHWs using the modified cetyltrimethyl ammonium bromide (CTAB) method described in the CIMMYT laboratory protocols [28]. The high-throughput genotyping method DArTseq^TM^ [29] was applied to all samples in the Genetic Analysis Service for Agriculture (SAGA) at CIMMYT, Texcoco, Mexico.

Out of the complete set of 443 SHW lines, 438 were genotyped and used for GWAS [25,26]. A total of 67,436 markers were scored, out of which 50% (34,790) could be aligned to reference genomes. Quality control was carried out based on the minimum lack of alleles, resulting in 5800 markers to be used for GWAS. The reference genomes used in this study were Chinese Spring IWGSC RefSeq v1.0 genome assembly [30] and durum wheat (cv. Svevo) Ref Seq Rel. 1.0 [31], along with the reference genome of *Ae. tauschii* (v.4, 2017) [32].

### 2.3. Phenotypic Model for Disease Traits TS, SNB and SB

Before applying the genomic predictive model, we estimated the effects of the cultivars using a linear model for ordinal traits, where the response can take values on C ordered values, $y_{ij}\in \left\{1,\ldots ,C\right\}$. We used the probit link, and the probability of each observation belonging to each category is given by:

$$P\left(y_{ij}=c\right)=\Phi \left(\eta_{\mathrm{ij}}-\gamma_{c}\right)-\Phi \left(\eta_{\mathrm{ij}}-\gamma_{c-1}\right),$$

where $\Phi \left(\cdot \right)$ corresponds to the cumulative distribution function of a standard normal random variable $\eta_{\mathrm{ij}}=r_{j}+l_{i}$, which corresponds to the linear predictor, which includes the effect of replicates ($r_{j})$ and SHW cultivars $(l_{i}$), and $\gamma_{c}$ are threshold parameters, with $\gamma_{0}=-\infty ,\;\gamma_{c}\geq \gamma_{c-1},\;\gamma_{C}=\infty$. For further details about the threshold model, see Gianola [33]. Note that the effects of the cultivars ($l_{i}$) or adjusted means computed are considered as response variables in the genomic prediction models.

### 2.4. Genomic Prediction Models

#### 2.4.1. Whole Genome Models

In these models, the genome markers are considered as a whole, without separating the three sub-genomes (A, B, and C). The first model is model G, which accounts for additive effects, and the second model is model GI, which includes additive effects and epistatic effects, similar to those presented by Santantonio [1].

***Model G***

This is the traditional general model for additive effects used in genomic prediction and selection, known as GBLUP:

(1) $$y=\mu 1+Zg_{G}+\varepsilon$$

where vector y represents the phenotypic observations, the overall mean of the intercept is μ, matrix Z is an incidence matrix that relates the observations to the genomic random effects ($g_{G}$). These are assumed to follow a normal distribution $g_{G}\sim N\left(0,\sigma_{g_{G}}^{2}G_{G}\right)$ with a mean of zero, a variance component $\sigma_{g_{G}}^{2}$, and a known covariance matrix $G_{G}$ constructed with the (*p*) genomic markers $X_{G}$ according to VanRaden [17] and adjusted by López-Cruz [34] to achieve a mean of one in the diagonal and zero in the off diagonal on $G_{G}$ using a marker matrix $X_{G}$ scaled with a mean of zero and variance equal to one.

(2) $$G_{G}=\frac{X_{G}X_{G}^{\prime }}{p}$$

Finally, the random errors of model ε are assumed to follow a normal distribution with a mean of zero and homogeneous variance $\sigma_{\varepsilon }^{2}$.

***Model GI***

Model GI considers additive and the interaction of additive × additive epistatic effects:

(3) $$y=\mu 1+Zg_{G}+Zg_{GI}+\varepsilon .$$

The interaction of random additive × additive effect $Zg_{GI}$ aims to capture the epistatic effects and is assumed to follow a normal distribution $g_{GI}\sim N\left(0,\sigma_{g_{GI}}^{2}G_{G}\#G_{G}\right)$, with a variance component to be estimated as $\sigma_{g_{GI}}^{2}$, and a known variance covariance matrix constructed as the Hadamard (#) of matrix $G_{G}$ containing the additive relationship information. The rest of the elements of model GI were already defined.

#### 2.4.2. Sub-Genome Models

In these models, the markers are divided by sub-genomes A, B, and D to capture their additive and epistatic effects within and between sub-genomes. The first model, ABD, is similar to the additive model proposed by Santantonio [1], and the second, ABDI, differs from Santantonio [1] by incorporating inter-sub-genome interactions. To achieve this, it is essential to have a reference map of the sub-genomes to separate markers for each sub-genome. In this study, markers are appended as $X_{A},X_{B},X_{D}$ for each sub-genome (refer to the Data Availability Statement section for details).

***Model ABD***

This model considers the additive effects of each sub-genome:

(4) $$y=\mu 1+Zg_{A}+Zg_{B}+Zg_{D}+\varepsilon$$

where $g_{A},\;g_{B},g_{D}$ denote the additive random effects of sub-genome A, B, and D, and it is assumed that they follow a normal distribution $g_{A}\sim N\left(0,\sigma_{g_{A}}^{2}G_{A}\right)$, $g_{B}\sim N\left(0,\sigma_{g_{B}}^{2}G_{B}\right)$, and $g_{D}\sim N\left(0,\sigma_{g_{D}}^{2}G_{D}\right)$ with variance covariance matrices $G_{A}$, $G_{B}$, and $G_{D}$ constructed with the markers by sub-genome $X_{A},X_{B},X_{D}$ as in (2), that is, $G_{A}=\frac{X_{A}X_{A}^{\prime }}{p_{A}};\;G_{B}=\frac{X_{B}X_{B}^{\prime }}{p_{B}};G_{D}=\frac{X_{D}X_{D}^{\prime }}{p_{D}}$ where $p_{A},p_{B},p_{D}$ represent the number of markers in each sub-genome.

***Model ABDI***

In this model, the additive effects within each sub-genome, the interaction effects within sub-genomes, and the interaction effects between sub-genomes are represented by:

(5) $$y=\mu 1+Zg_{A}+Zg_{B}+Zg_{D}+Zg_{AA}+Zg_{BB}+Zg_{DD}+Zg_{AB}+Zg_{AD}+Zg_{BD}+\varepsilon$$

where random effects $g_{A},\;g_{B},g_{D}$ represent the additive genomic effects within each sub-genome, individually defined in the previous model. The random interaction effects within each sub-genome are represented by vectors $g_{AA},g_{BB},g_{DD}$, which follow a normal distribution like the GI model, i.e., $g_{AA}\sim N\left(0,\sigma_{g_{AA}}^{2}G_{A}\#G_{A}\right),\;g_{BB}\sim N\left(0,\sigma_{g_{BB}}^{2}G_{B}\#G_{B}\right)$, and $g_{DD}\sim N\left(0,\sigma_{g_{DD}}^{2}G_{D}\#G_{D}\right)$. Finally, the random interaction effects between sub-genomes are represented by vectors $g_{AB},g_{AD},g_{BD}$, which are assumed to follow a normal distribution, i.e., $g_{AB}\sim N\left(0,\sigma_{g_{AB}}^{2}G_{A}\#G_{B}\right),g_{AD}\sim N\left(0,\sigma_{g_{AD}}^{2}G_{A}\#G_{D}\right)$, and $g_{BD}\sim N\left(0,\sigma_{g_{BD}}^{2}G_{B}\#G_{D}\right)$.

### 2.5. Cross-Validation Schemes

Three types of prediction problems were assessed (CV1, CV2, and CV3), like those used by Basnet [35] and Dreisigacker [19]. In each case, 50 random samples were obtained for the training (Training) and testing (Testing) groups to make predictions for the test group. For each sample, the Pearson correlation between the predicted values and the test values was calculated. The means of the correlations and their standard errors are reported. The 50 random samples were composed of five folds according to the type of cross-validation (CV), with 10 repetitions of this process.

We performed a CV1 random cross-validation analysis, which considered that certain proportion SHWs were assessed for disease resistance, whereas for others, SHW phenotypic values are unobserved (missing). CV1 reflected the problem breeders face of usually not having the full capacity to evaluate all possible cultivars (germplasm) for all types of target traits. The cross-validation scheme CV2 assessed the problem of predicting an SHW whose DW parent has not yet been observed in any SHW combination. The training set included all the SHW lines obtained when using 80% of the DW lines crossed with *Ae. tauschii* and predicting the remaining 20% of the DW parents crossed with the *Ae. tauschii*. The cross-validation scheme CV3 was similar to CV2; we performed cross-validation assigning the *Ae. tauschii* wheat parents to folds. Here, the training set included all the SHWs obtained when using 80% of the *Ae. tauschii* crossed with durum wheat and predicting the remaining 20% of the SHW. This CV scheme reflected the problem of predicting SHW using DW parents whose crosses with any of the *Ae. tauschii* accessions have not yet observed.

In each case, 50 random samples were obtained for training (Training) and testing (Testing) groups to make predictions for the testing group and calculate the Pearson correlation between the predicted values and the observed values (Testing group) for each sample. The 50 random samples were organized into five folds according to the type of cross-validation (CV1, CV2, or CV3), with 10 repetitions of this process.

### 2.6. Software

Models were fitted using the R library BGLR 1.1.1 (Pérez and de los Campos [36] with 100,000 iterations and a burn in of 10,000 and a thinning of 10, to minimize random errors as much as possible.

## 3. Results

### 3.1. Estimated Variance Components for the Different Traits and Statistical Models

In Table 1, the estimated genomic and residual variance components for each of the statistical models G, GI, ABD, and ABDI are shown for the analyzed traits TS, SNB, and SB. Considering the residual variances, one observes the pattern of the residual variance being reduced when transitioning from G to ABD, that is, when splitting the overall additive variance into the three sub-genome variances. This is true for TS, where the residual variance decreases from 0.330 to 0.326, as well as for SNB, where it decreases from 0.403 to 0.396, and for SB (from 0.535 to 0.503).

When considering model GI, which includes a whole-genome additive effect and a whole-genome interaction effect, the residual variance is reduced further to 0.261, 0.259, and 0.416 for the traits TS, SNB, and SB respectively. Here, for the transition from G to GI, which means adding one term describing pairwise marker interactions, the reduction in the residual variance is between 20% and 35%. This observation indicates that the structure of the phenotypic data can be captured better when including statistical epistasis in the model. The results indicate that epistasis makes a significant contribution to explaining genomic variation in the inbred wheat populations, accounting for approximately 50% in SNB and SB, and only 29% for TS.

When splitting additive effects and interactions according to sub-genomes in the ABDI model, the residual variance components are further reduced to 0.234 (TS), 0.235 (SNB), and 0.353 (SB), which means that overall, the residual variance is reduced by 29% (TS), 42% (SNB), and 39% (SB) compared to model G. We also observe (1) that the contributions of intra- and inter-sub-genome interactions of the D sub-genome in epistasis are 49% (TS), 60% (SNB), and 60% (SB), and (2) that the contributions of the intra-sub-genome interactions (AA, BB, and DD) are approximately 51%, 49%, and 54% of the epistasis for the diseases TS, SNB, and SB, respectively.

**Figure 1:**
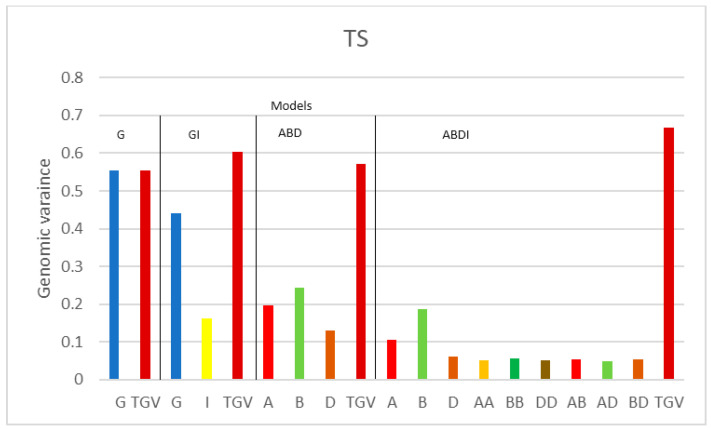
Genomic variance components of SHW for disease TS for the four models G, GI, ABD, and ABDI. G represents the additive genetic variance of the SHW, TGV denotes the total genetic variance of SHW, and I is the additive × additive interaction (epistasis) variance of the SHW. A, B, and D are the additive genetic variances of each sub-genome; AA, BB, and DD are the epistasis variances within sub-genomes A, B, and C, and AB, AD, and BD are the epistasis variance between sub-genomes A, B, and D.

**Figure 2:**
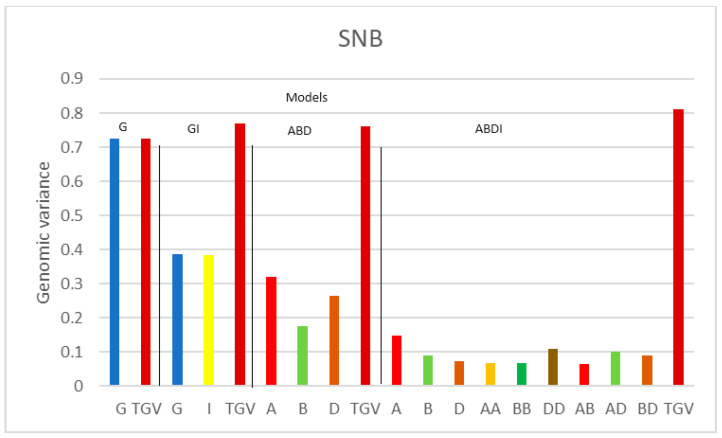
Genomic variance components of SHW for disease SNB for the four models G, GI, ABD, and ABDI. G represents the additive genetic variance of the SHW, TGV denotes the total genetic variance of SHW, and I is the additive × additive interaction (epistasis) variance of the SHW. A, B, and D are the additive genetic variances for each sub-genome; AA, BB, and DD are the epistasis interaction variances within sub-genomes A, B, and C, and AB, AD, and BD are the epistasis interaction variances between sub-genomes A, B, and D.

**Figure 3:**
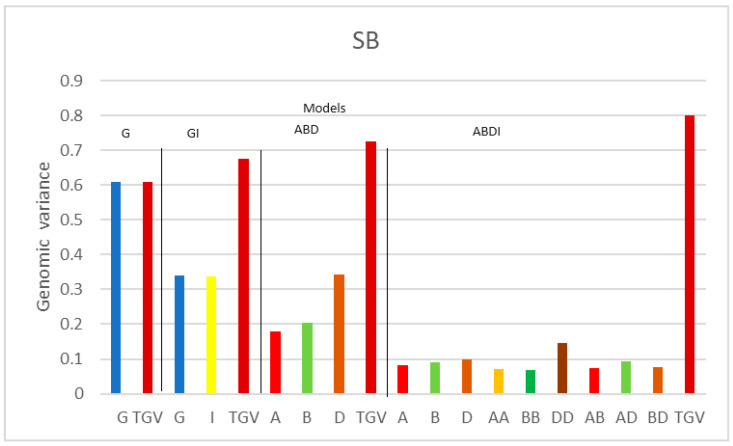
Genomic variance components of SHW for disease SB for the four models G, GI, ABD, and ABDI. G represents the additive genetic variance of the SHW, TGV denotes the total genetic variance of SHW, and I is the additive × additive interaction (epistasis) variance of the SHW. A, B, and D are the additive genetic variances for each sub-genome; AA, BB, and DD are the epistasis interaction variances within sub-genomes A, B, and C, and AB, AD, and BD are the epistasis interaction variances between sub-genomes A, B, and D.

Figure 1, Figure 2 and Figure 3 illustrate the genomic variance components, displaying the components of one trait at a time for each model, along with the total genomic variance (TGV). The estimated variance components may vary when using different definitions of genomic (epistatic) relationship matrices. Therefore, for comparisons, we mainly focus on a certain class of effects, such as on the variance components of the three additive sub-genome matrices, or on comparisons across traits, for which the analyses are based on the same matrices.

Considering the trait TS (Figure 1), we see an expected behavior for models G and GI: When introducing the epistatic effects, the additive variance component of G is reduced to around 4/5 of its size in GI (from 0.555 to 0.440). The main part of the estimated variance component remains as “additive variance”, yet 1/5 of the additive variance of G is captured by the interaction effects in GI. Moreover, we see that the additive variance component related to the D genome is smaller than the additive variance components of A and B in both ABD and ABDI. The variance components attributed to the different pairwise interactions of the ABDI model are on a similar level. These results suggest that the A and B sub-genomes are more relevant than the D sub-genome for the trait TS.

When considering the variance components for SNB (Figure 2), we see a different pattern. First, the instruction of an epistatic relationship matrix (from G to GI) reduces the estimated additive variance component from 0.724 to 0.386, which means that more than 55% of the additive variance component in G is attributed to interactions when including both additive effects and pairwise interaction effects in model GI. Moreover, comparing the additive variance components of the three sub-genomes, the variance component of sub-genome B is smaller than that of A and D in the ABD model. Also, the within-sub-genome interaction of sub-genome D stands out of the epistatic interactions and even exceeds the additive variance components. These results suggest that the D sub-genome plays a more important role for SNB.

The tendency described for SNB of (i) sub-genome D having a more important role and (ii) statistical interactions being more relevant can also be observed for SB (Figure 3). Here, once again, the additive variance component of G drops from 0.608 to 0.339 when using model GI. Moreover, the sub-genome additive variance of sub-genome D is higher than for the other sub-genomes in model ABD, which is mainly captured by within-D interactions in the ABDI model.

### 3.2. Genomic Prediction

The average predictive abilities and their standard error (SE) per trait and model are shown in Table 2. As a first observation, it is evident that for each trait and each type of CV, the highest predictive ability is always obtained by a model including interactions, that is, model GI or ABDI. Moreover, out of the nine traits by CV combinations, GI has the highest predictive ability in three cases, compared to seven cases in which ABDI showed the highest predictive ability (for TS and CV1, both models GI and ABDI showed the same predictive ability). These results indicate that the inclusion of statistical interaction epistatic effects not only fits the data better, as indicated by the reduction in the error variances as described earlier, but it also leads to a higher predictive ability. The highest predictions for two traits (TS and SB) for CV1 and CV3 correspond to model ABDI (0.724 and 0.715 for CV1 and CV3, respectively, for disease TS; 0.506 and 0.500 for CV1 and CV3, respectively, for disease SB).

Moreover, we see that for almost all model and trait combinations, CV2, that is, when the DW parent has not been included in any combination in the training set, has the lowest predictive ability compared to CV1 and CV3. This may be related to the relevance of the A and B sub-genomes, but is more likely enhanced by the data structure, and reduces the training set more strongly when restricted to 80% of the DW parents (recall that DW parents were used in between 1 and 54 crosses, whereas the *Ae. tuschii* lines were not used in more than 7 crosses).

Comparing the behavior of the predictive abilities across traits, we also see that for most models, CV1 has the highest predictive ability, the predictive ability of CV2 decreases, and the predictive ability of CV3 increases again. Figures visualize the average correlations (bars) and a one-standard-error interval (whiskers), organized by cross-validation type (CV1, CV2, or CV3), and within each figure, predictive results by trait and model.

**Figure 4:**
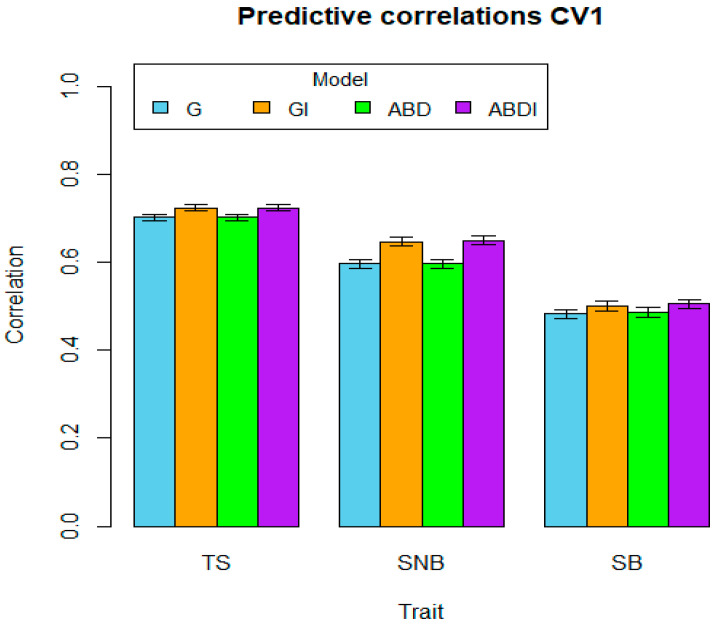
Genomic prediction ability of the four models G, GI, ABD, and ABDI for the three disease traits TS, SNB, and SB for random cross-validation CV1. The bars represent the mean of predictive correlations, and the whiskers represent distance intervals of one standard error from the mean.

For the trait TS, we can observe that in the GI model, the interaction component I (Figure 1, Table 1) has a relatively small proportion of influence (0.163) compared to the additive component G (0.406). This may explain why the average predictions (Table 2) for the GI model (0.724) are not as high as those for the G model (0.702) for CV1. In the ABD model, the major variance components correspond to the A and B sub-genomes, with 0.196 and 0.244, respectively, while the D sub-genome only has 0.131. This behavior is different from the other traits in the ABD model. In the ABDI model, the additive components of the A and B sub-genomes show significant influence at similar magnitudes (0.105 and 0.187, respectively), while the epistatic components (AA, BB, DD, AB, AD, and BD) are similar and close to 0.05, reducing the residual component to 0.234. This may explain the increase in prediction for the ABDI model to 0.724 for CV1 (Figure 4). The intervals in the figures represent the standard error (SE) for each model and thus establish the significant differences between the genomic ability of the models. The bars represent the mean of the predictive correlations, and the whiskers denote distance intervals of one standard error from the mean, which aligns with the concept of a box plot where the bars signify the mean or median, and the whiskers represent a certain range or dispersion, typically one standard error.

**Figure 5:**
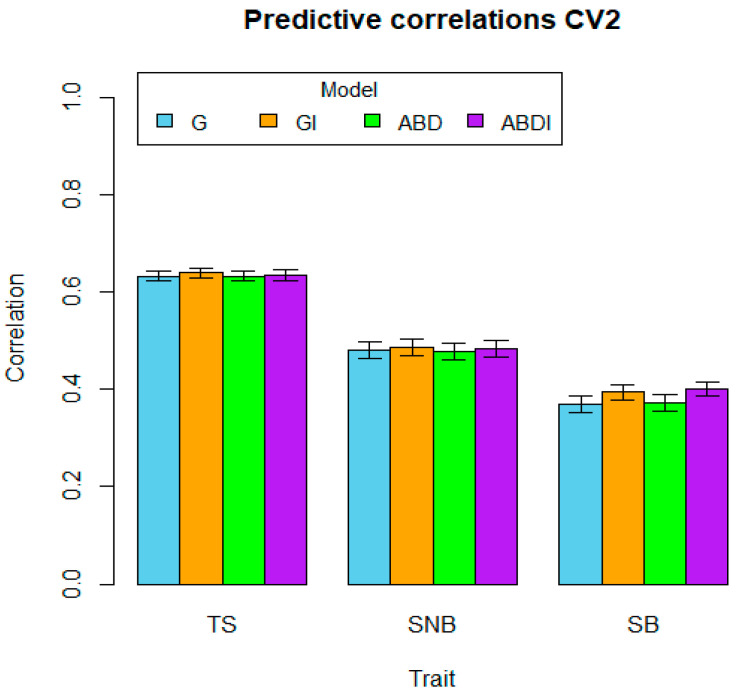
Genomic prediction ability of the four models G, GI, ABD, and ABDI for the three disease traits TS, SNB, and SB for random cross-validation CV2. The bars represent the mean of predictive correlations, and the whiskers represent distance intervals of one standard error from the mean.

For the SNB trait, the interaction component of the GI model is similar (0.383) to the additive component G (0.386) (Table 1, Figure 2), reducing the residual component to 0.259 compared to 0.403 for the G model. This seems to explain the better prediction of the GI model (Table 2, Figure 5) with an average of 0.647, compared to 0.597 for the additive G model. In the ABD model, additive components A and D have higher values of 0.321 and 0.265, respectively, compared to 0.176 for B. In the ABDI model, the additive component A and epistatic components DD, AD, and BD have higher values of 0.109, 0.100, and 0.091, although the intra-sub-genome interaction (epistasis) of AA and AB is low, 0.067 and 0.065, respectively, showing the best average predictive correlations of 0.650 for CV1 (Table 2, Figure 5)

**Figure 6:**
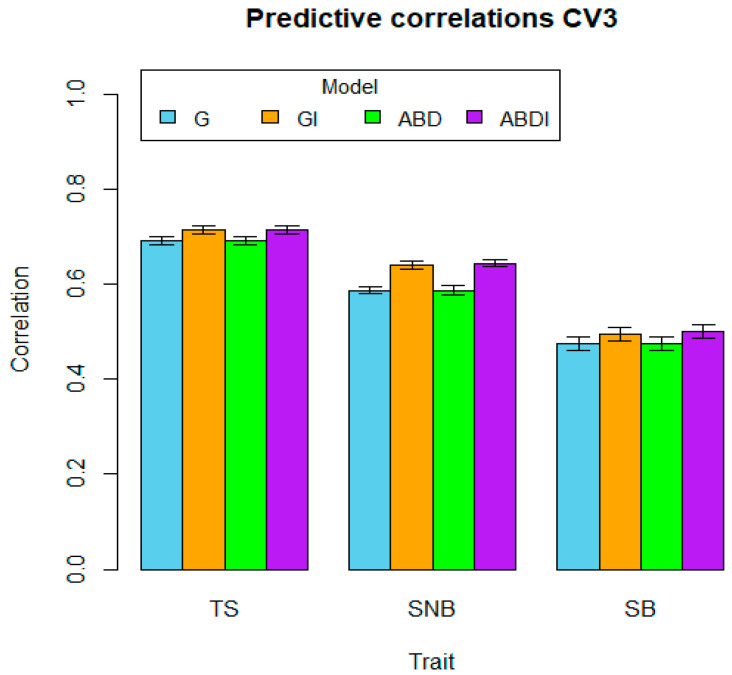
Genomic prediction ability of the four models G, GI, ABD, and ABDI for the three disease traits TS, SNB, and SB for random cross-validation CV3. The bars represent the mean of predictive correlations, and the whiskers represent distance intervals of one standard error from the mean.

For the SB trait, the global epistatic component of the GI model is similar (0.337) to the additive component (0.339). Similarly, in the other cases, the average predictive correlations of the GI model are 0.500, which is higher than the G model’s 0.482. The influence of the D sub-genome is very pronounced in the ABD model with a component of 0.343, and the DD interaction component also stands out in the ABDI model with an estimated value of 0.146 (Figure 3), followed by, in order of importance, the AD, BD, AB, AA, and BB interactions, with values of 0.092, 0.077, 0.075, 0.07, and 0.069, respectively. The latter model has an average prediction of 0.506, making it the best among the models for CV1 (Figure 6).

## 4. Discussion

### 4.1. Activation of Genes from Sub-Genome D with Those of Sub-Genome A and B

With the increasing number of diseases affecting cultivated wheat plants, the option of developing resistance SHW lines has been widely used. Lozano-Ramirez [26] studied significant marker–trait association from a diverse collection of 443 SHW lines, and 41 significant markers and a range of SHW lines with high SB resistance were identified. In the analysis, the authors identified a subset of markers and SHW lines that are more suitable for future breeding and pre-breeding activities. Lozano-Ramirez [26] identified 41 significant markers related to SB resistance, distributed on 15 wheat chromosomes, and many of them were novel. The authors were able to identify highly resistant SHWs with the most resistance alleles of the significant markers, and this can be used in future wheat-breeding programs. Intrinsically, the genes from sub-genome D contributed by *Ae. tauschii* activate (interact, epistatic) genes from sub-genomes A and B. Chu [37] found that the expression of several resistance genes in DW is suppressed but becomes activated when DW is crossed with *Ae. tauschii*. This type of activation function of resistance genes existing in the A and B sub-genomes when joining with the D sub-genome could be due to epistasis (inter-locus interaction) (epistasis effects).

Lozano-Ramirez [25] identified new sources of genetic resistance in SHWs that can provide enhanced resistance to TS disease in elite bread wheat SHW at CIMMYT. The authors found around 30 significant marker–trait associations, of which some fell into one common QTL. Lozano-Ramirez et al. [25] found significant epistatic effects, (1) mainly activation interactions driven by the D sub-genome of hexaploid wheat; (2) epistasis effects increased resistance in the SHW in comparison to their direct susceptible DW parents. The authors concluded that a few of their MTAs were novel and significantly increase the number of resistance sources, specifically derived from *Ae. tauschii* accessions in the D genome.

### 4.2. Study of Epistasis of Sub-Genome A, B, and D Genes

VanRaden’s [17] method to compute the additive genomic relationship matrix is used as in López-Cruz [34], with markers scaled to a mean of one and a variance of zero, with the intention that the variance components represent the proportion of their contribution, and the sum of these approximates to one. To build the inter-genomic epistasis, we use Hadamard products [21,22].

In the statistical models used, it is assumed that each marker is an independent covariate. In this sense, grouping markers to form relationship matrices for analysis is accepted, as in Akdemir [38] and in Santantonio [1], which partitions the genomic covariance matrices according to their A, B, and D sub-genomes, assuming they are independent, and it is possible to form multi-components that are also statistically assumed to be independent from each other.

The desirable outcome is for the random components of the additive and epistatic effects to be completely independent and uncorrelated, so that the additive variation obtained in the G and ABD models is not reduced or combined with the variation of epistatic effects in the GI and ABDI models, respectively, masking the exact value of the variation of epistatic effects. However, total independence and a lack of correlation cannot be guaranteed between markers or between additive and epistatic effects, as pointed out by Vitezica [39]. This may be due to the strong linkage disequilibrium (LD) characteristic of wheat, which generates non-independence between loci. Vitezica [39] conducted a simulation where the zero correlation between additive and epistatic effects disappeared with a strong LD compared to the linkage equilibrium, where the correlation was close to zero. Like this work, where the additive variation obtained in the G and ABD models was reduced and combined with the epistatic components of GI and ABDI, it was also reported in Santantonio [1], although this author did not consider the inter-sub-genome epistasis.

The simple way to identify epistatic variation from G to GI is by calculating the difference in variation in residuals from G to GI. However, for ABD to ABDI, it represents the total epistatic variation. The results of this study on three diseases, TS, SNB, and SB, show the importance of the D sub-genome in modeling genomic variation, which seems to be specific to the conception of the SHW [19]. In other studies, such as those presented in González-Diéguez [40], similar results were obtained regarding variance components for disease traits. However, in yield traits, their findings suggested that sub-genomes A and B were predominantly dominant.

The ABD additive model fits better than the G model because the ABD model has three variances that weigh each of the sub-genomes and considers three weights for the markers, allowing greater flexibility. In contrast, the G model assumes that all markers have the same weight. The ABD model better represents the structure of the phenotypes; however, in the results, there is no statistical evidence that this fact improves the predictions of unobserved lines. The comparison is similar to ABDI, which better represents the phenotypic variation compared to the ABD model, and the results are better without sufficient statistical evidence.

## 5. Conclusions

In the analysis of variance components, it is observed that additive epistasis is more pronounced in SNB (50%) and SB (50%) compared to TS (27%). The influence of intra- and inter-sub-genome interactions of the D sub-genome on the contribution of epistasis is significant in SNB (60%), SB (60%), and TS (49%). This indicates that the influence of sub-genome D on disease traits favors epistasis and enhances predictive ability, with an increase from the G model to the ABDI model for CV1 by 1%, 9%, and 5% for TS, SNB, and SB, respectively. These results, consistent with other studies, underscore the importance of the D sub-genome in disease traits and suggest a potential application to be explored in the future regarding the selection of parental crosses based on sub-genomes.

## Figures and Tables

**Table 1:** Variance components for traits TS, SNB, and SB for models G, GI, ABD, and ABDI. Variance of the residual (Res).

		Variance Components											
TRAIT	Model	$\sigma_{G}^{2}$	$\sigma_{GI}^{2}$	$\sigma_{GA}^{2}$	$\sigma_{GB}^{2}$	$\sigma_{GD}^{2}$	$\sigma_{GAA}^{2}$	$\sigma_{GBB}^{2}$	$\sigma_{GDD}^{2}$	$\sigma_{GAB}^{2}$	$\sigma_{GAD}^{2}$	$\sigma_{GBD}^{2}$	Res
TS	G	0.555											0.330
	GI	0.440	0.163										0.261
	ABD			0.196	0.244	0.131							0.326
	ABDI			0.105	0.187	0.061	0.052	0.056	0.052	0.053	0.048	0.054	0.234
SNB	G	0.724											0.403
	GI	0.386	0.383										0.259
	ABD			0.321	0.176	0.265							0.396
	ABDI			0.148	0.091	0.072	0.067	0.068	0.109	0.065	0.100	0.091	0.235
SB	G	0.608											0.535
	GI	0.339	0.337										0.416
	ABD			0.179	0.205	0.343							0.503
	ABDI			0.081	0.090	0.100	0.072	0.069	0.146	0.075	0.092	0.077	0.353

**Table 2:** Genomic prediction ability represented by the average (mean) correlation between observed and predicted values from models G, GI, ABD, and ABDI for five-fold random cross-validation for cross-validations CV1, CV2, and CV3 (standard errors, SEs, are in parenthesis). The best means of models for each disease trait and cross-validation are given in bold for each trait.

Traits		CV1				CV2				CV3			
		G	GI	ABD	ABDI	G	GI	ABD	ABDI	G	GI	ABD	ABDI
TS	Mean	0.702	**0.724**	0.702	**0.724**	0.632	**0.639**	0.632	0.635	0.691	0.714	0.691	**0.715**
	SE	0.007	0.007	0.007	0.007	0.010	0.011	0.010	0.011	0.009	0.009	0.009	0.009
SNB	Mean	0.597	0.647	0.596	**0.650**	0.481	**0.487**	0.479	0.484	0.587	0.641	0.587	**0.644**
	SE	0.010	0.009	0.009	0.009	0.017	0.017	0.017	0.017	0.008	0.008	0.009	0.008
SB	Mean	0.482	0.500	0.486	**0.506**	0.370	0.394	0.371	**0.401**	0.475	0.495	0.476	**0.500**
	SE	0.011	0.011	0.011	0.010	0.017	0.015	0.017	0.014	0.014	0.014	0.014	0.014

## Data Availability

The phenotypic and genotypic (marker) for the 5 data sets (TS, SNB, and SB) can be found in the following link: https://hdl.handle.net/11529/10548948.
